# Thermoresponsive Behavior, Degradation, and Bioactivity of Nanohydroxyapatite on Graphene Oxide Nanoscroll-Enhanced Poly(N-isopropylacrylamide)-Based Scaffolds

**DOI:** 10.3390/polym17152014

**Published:** 2025-07-23

**Authors:** Lillian Tsitsi Mambiri, Riley Guillory, Dilip Depan

**Affiliations:** Chemical Engineering Department, Institute for Materials Research and Innovation, University of Louisiana at Lafayette, Lafayette, LA 70504, USA; lillian.mambiri1@louisiana.edu (L.T.M.); riley.guillory1@louisiana.edu (R.G.)

**Keywords:** thermoresponsive, oxidative degradation, stereolithography, nanohydroxyapatite, antioxidant, graphene oxide nanoscrolls

## Abstract

Osteoarthritis and metastatic bone cancers create pathological oxidative environments characterized by elevated reactive oxygen species (ROS). ROS impair bone regeneration by degrading the scaffold and suppressing mineralization. To address these challenges, we fabricated thermoresponsive scaffolds based on poly(N-isopropylacrylamide) (PNIPAAm) incorporating in situ-grown nanohydroxyapatite on graphene oxide nanoscrolls (nHA-GONS) using stereolithography (SLA). Three scaffold formulations were studied: pure PNIPAAm (PNP), PNIPAAm with 5 wt.% nHA-GONS (P5G), and PNIPAAm with 5 wt.% nHA-GONS reinforced with polycaprolactone (PCL) microspheres (PN5GP). Each scaffold was evaluated for (i) swelling and lower critical solution temperature (LCST) using differential scanning calorimetry (DSC); (ii) oxidative degradation assessed using Fourier-transform infrared spectroscopy (FTIR), mass loss, and antioxidant assays; and (iii) mineralization and morphology via immersion in simulated body fluid followed by microscopy. The PN5GP and P5G scaffolds demonstrated reversible swelling, sustained antioxidant activity, and enhanced calcium deposition, which enable redox stability and mineralization under oxidative environments, critical for scaffold functionality in bone repair. PNP scaffolds exhibited copper accumulation, while PN5GP suffered from accelerated mass loss driven by the PCL phase. These findings identify the P5G formulation as a promising scaffold. This study introduces a quantitative framework that enables the predictive design of oxidation-resilient scaffolds.

## 1. Introduction

Bone tissue regeneration demands scaffolds that replicate the hierarchical organic–inorganic structure of native bone while addressing dynamic physiological challenges such as oxidative stress, enzymatic degradation, and temperature fluctuations [1]. Polycaprolactone (PCL) composites are widely used for their mechanical robustness but suffer from diffusion-driven degradation in aqueous environments, leading to premature structural failure [2,3]. Compounding this issue, aging populations and chronic diseases create oxidative microenvironments that further degrade scaffold performance [1,4]. This increase in disease burden inspires the need for scaffold design improvement that can withstand complex physiological conditions.

Oxidative stress, driven by reactive oxygen species (ROS), presents a formidable barrier to effective bone tissue regeneration, particularly in oxidative microenvironments associated with aging, osteoporosis, and chronic inflammatory conditions [5]. Hydrogen peroxide (H_2_O_2_), a key component and driver of oxidative stress, detrimentally influences scaffold performance through multiple mechanisms. It accelerates degradation kinetics, leading to a reduction in mechanical strength, while simultaneously suppressing osteogenic differentiation, as evidenced by a decrease in alkaline phosphatase (ALP) activity [6]. Furthermore, H_2_O_2_ disrupts scaffold–tissue integration, reducing bone-to-scaffold contact, and induces a pro-inflammatory microenvironment marked by an increase in tumor necrosis factor-alpha levels [7,8]. ROS-mediated chain scission and oxidative cleavage accelerate scaffold breakdown, undermining mechanical integrity, bioactivity, and long-term functionality. Biomineralization, the process by which bone-like mineral is formed, is heavily affected by oxidative stress [9]. Since biomineralization is critical for restoring mechanical function and facilitating scaffold integration, understanding how oxidative stress impairs this process is essential.

Under normal conditions, scaffolds provide structured surfaces with functional groups (−COOH, −OH, −NH_2_) and crystalline domains that template hydroxyapatite formation. However, ROS oxidize these surface groups, reducing their ability to coordinate calcium and phosphate ions, while also degrading polymeric scaffolds, leading to loss of crystallinity and disordered nucleation sites [9]. Additionally, oxidative stress disrupts calcium and phosphate homeostasis by generating oxidized byproducts that chelate Ca^2+^ or create acidic microenvironments, inhibiting mineral deposition even in supersaturated conditions [10]. The accelerated, uncontrolled degradation of scaffolds under ROS further exacerbates the problem, releasing acidic byproducts that destabilize local pH and autocatalyze degradation [10]. The absence of biomineralization accelerates scaffold failure on multiple fronts: mechanical collapse occurs within 4–6 weeks due to rapid degradation, biological dysfunction manifests by 8–12 weeks as osteogenic activity ceases, and clinical complications such as fibrous encapsulation arise from poor integration [11].

To counteract these effects, antioxidant-functionalized scaffolds have been developed to scavenge ROS, maintain surface integrity, and stabilize the redox microenvironment, thereby promoting orderly mineral nucleation and growth. Graphene oxide nanoscrolls (GONS) offer a promising solution: they impart shear-thinning behavior for improved printability, exhibit antioxidant activity to scavenge ROS, and act as an effective interfacial bridge that templates in situ nanohydroxyapatite (nHA) growth, promoting mineralization [2,12]. Recent efforts have explored introducing dynamic functionality through thermoresponsive polymers, particularly poly N-isopropyl acrylamide (PNIPAAm) [13,14], and reinforcing with bioactive fillers, but challenges remain in understanding the effects on sustained antioxidant protection and mineralization under oxidative stress.

Additive manufacturing techniques, particularly stereolithography (SLA), offer the precision to fabricate PNIPAAm-based scaffolds with bone-mimetic architectures. Furthermore, SLA allows the synthesis of resins with the multifunctionality needed to achieve (1) structural stability, (2) stimuli-responsive degradation control, and (3) oxidative resistance [5,15,16]. Notably, SLA processing occurs under mild photopolymerization conditions that preserve PNIPAAm’s properties without inducing thermal or chemical degradation, making it a suitable platform for maintaining polymer integrity during scaffold fabrication.

PNIPAAm exhibits a lower critical solution temperature (LCST) of around 32 °C, rendering it hydrophilic and swollen below this threshold, and hydrophobic and collapsed above it [13,17]. This significant volume change can modulate degradation by limiting water diffusion into the polymer network under physiological temperatures around 37 °C [14,17,18]. On its own, however, PNIPAAm has insufficient mechanical strength, necessitating the use of reinforcing materials such as PCL and GONS.

This study aims to fabricate and characterize SLA-printed PNIPAAm/PCL scaffolds incorporating in situ-grown nHA on GONS to test whether (i) GONS’ intrinsic antioxidant activity preserves scaffold integrity and LCST behavior in 5–10 µM H_2_O_2_, and (ii) their scroll morphology nucleates orderly hydroxyapatite under oxidative stress, thereby achieving structurally stable, bioactive scaffolds for applications in bone regeneration.

Unlike earlier PNIPAAm or PCL composites that address printability, ROS scavenging, or bioactivity in isolation, this work integrates all three functions in a single SLA-printed construct by leveraging the redox and templating properties of GONS. The study, therefore, establishes a design framework for dynamic scaffolds that remain biofunctional, antioxidative, and mineralization-competent throughout the early bone-healing window in vitro. Additionally, most PNIPAAm studies examine LCST-driven swelling under neutral conditions; pairing it with ROS degradation and GONS is still uncommon. This study delivers the first quantitative design framework that couples GONS and ROS-scavenging kinetics to LCST drift, hydrolytic–oxidative chain scission, and nHA nucleation, enabling the predictive engineering of oxidation-resilient, thermoresponsive scaffolds.

## 2. Materials and Methods

N-isopropylacrylamide (NIPAAm), N, N′-methylene-bis(acrylamide), bis(diphenyl phosphine oxide), polycaprolactone (PCL, average Mn 14,000), calcium nitrate tetrahydrate (Ca(NO_3_)_2_·4H_2_O), lysozyme, diammonium phosphate ((NH_4_)_2_HPO_4_), hydrogen peroxide (H_2_O_2_), copper (II) sulfate (CuSO_4_), sodium chloride (NaCl), sodium bicarbonate (NaHCO_3_), potassium chloride (KCl), magnesium chloride hexahydrate (MgCl_2_·6H_2_O), sodium sulfate (Na_2_SO_4_), calcium chloride dihydrate (CaCl_2_·2H_2_O), and sodium hydrogen phosphate (Na_2_HPO_4_) were purchased from Sigma-Aldrich, Milwaukee, WI, USA. Graphene oxide (GO) aqueous dispersion (5 mg/mL) was obtained from Goographene, Merrifield, VA, USA. Phosphate-buffered saline (PBS), sodium L-lactate (C_3_H_5_NaO_3_), ammonium hydroxide (NH_4_OH), and lactic acid (C_3_H_6_O_3_) were acquired from Thermo Fisher Scientific, Waltham, MA, USA.

**Scheme 1:**
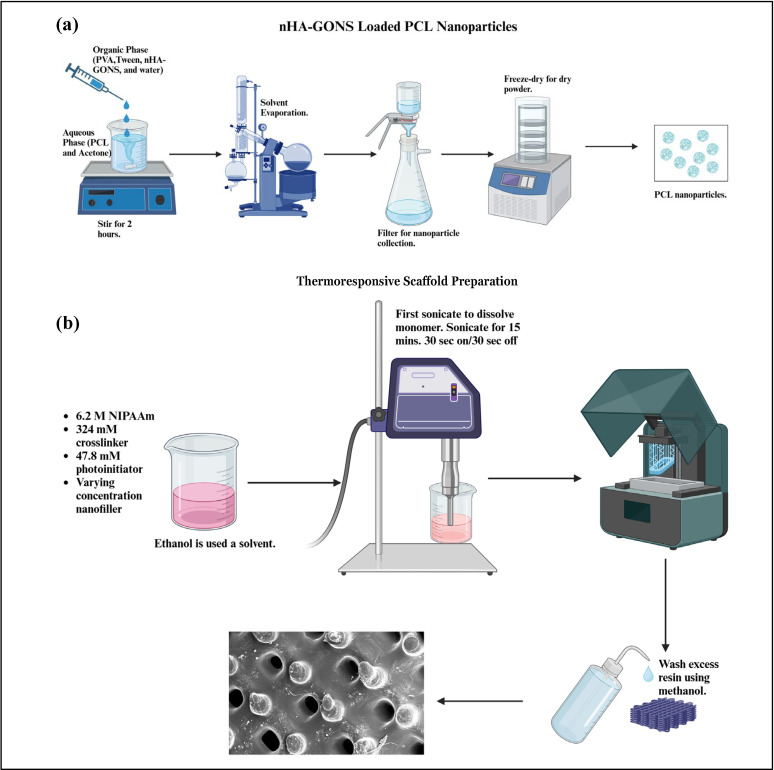
(**a**) Fabrication of PCL nanoparticles loaded with GONS, and (**b**) fabrication of 3D-printed thermoresponsive scaffolds.

nHA was synthesized on GONS using a wet precipitation method described in our earlier work. PCL nanoparticles were prepared by dissolving PCL in acetone (40 °C, 10 min) and sonicating for 30 min. This organic phase was added dropwise into an aqueous phase of PVA (95 °C, 12 h) and polysorbate (stirred 12 h). After 3 h of stirring, nanoparticles were purified by rotary evaporation, centrifugation, and freeze-drying. For composite nanoparticles, nHA-GONS was premixed into the organic phase at a 1:1 PCL:nHA-GONS weight ratio (Scheme 1a). A solution of 6.2 M NIPAAm was prepared in ethanol under continuous stirring until fully dissolved [19,20]. To this, 324 mM N, N′-Methylene-bis(acrylamide) (cross-linker), and 47.8 mM bis(diphenyl phosphine oxide) (photoinitiator) were added, and the mixture was stirred until homogeneous. The solution was stirred thoroughly until uniform (Scheme 1b). For this study, three groups of PNIPAAm-based resin samples were developed with varying compositions. Three sample formulations were prepared via stereolithography and named pure PNIPAAm (PNP), PNIPAAm + 5 wt.% nHA-GONS (P5G), and PNIPAAm + 5 wt.% nHA-GONS + PCL microspheres (PN5GP).

### 2.1. Oxidative Degradation

Kokubo’s method was modified to prepare the lactate-modified simulated body fluid (LAC-SBF) [21,22]. SBF is a standardized medium replicating the ionic composition of human extracellular fluid, commonly utilized to assess scaffold degradation in vitro. To mimic oxidative stress associated with inflammatory and pathological conditions, hydrogen peroxide (H_2_O_2_) at concentrations of 5 mM with 50 µM CuSO_4_ was incorporated into the SBF. This approach enabled the study of scaffold stability and degradation pathways under physiologically relevant oxidative environments. The scaffolds were weighed and then incubated in the oxidative degradation medium for 1, 3, 7, 21, and 28 days. After incubation, the scaffolds were washed, dried, and weighed again. The weight loss of each scaffold was calculated according to Equation (1).

(1) $$\frac{W_{i}-W_{f}}{W_{i}}\times 100=W_{loss}(\%)$$

W_i_ = Initial weight

W_loss_ = Weight loss

W_f_ = Final weight

### 2.2. Thermal Behavior, Aqueous Stability, and Phase Transition

The thermoresponsive behavior of the scaffolds was analyzed using a Perkin Elmer 4000 DSC (Shelton, CT, USA). The samples were crushed, hydrated, and weighed in aluminum pans to achieve a mass between 15 and 20 mg. The samples were heated from −10 °C to 50 °C and subsequently cooled back to −10 °C at a rate of 2 °C/min for three cycles to assess the reversibility and LCST [20].

### 2.3. Chemical Analysis

All FTIR spectra were acquired using a LUMOS II Bruker FTIR microscope (Ettlingen, Germany) equipped with ATR, in the spectral range of 4000–650 cm^−1^, at a resolution of 8 cm^−1^, using 32 scans and constant ATR pressure to ensure consistent peak intensity comparisons across degradation time points [23].

### 2.4. Morphological and Elemental Analysis

A Thermo Scientific Scios 2 Dual-Beam scanning electron microscopy (SEM) system (Waltham, MA, USA) was used to analyze surface morphology at an accelerating voltage of 5 kV. Before SEM observation, the scaffolds were mounted on aluminum stubs and gold-sputtered with a layer thickness of 10 nm. A Thermo Scientific Ultradry Energy-Dispersive Spectrometry (EDS) system was used to quantify mineralization.

### 2.5. Antioxidant Behavior

The antioxidant potential of the P5G and PN5GP samples was tested through radical scavenging activity using a DPPH (2,2-diphenyl-1-picrylhydrazyl) assay [24]. An amount of 0.8 mL of 95% methanol was mixed with 0.2 mL of methanolic DPPH solution and was used as the control group. Then, PNP and PN5GP samples (weighing 2.00 ± 0.01 mg) were dissolved in 10 mL of methanol to make the sample solution. The mixture was thoroughly shaken at room temperature. Afterward, the samples were placed in a dark room for 30 min, and the absorbance at 517 nm was measured using a Fisher Unico 1000 spectrophotometer (Dayton, NJ, USA). The DPPH assay was conducted using a fixed 30 min incubation period; no additional time points were evaluated in this study. The absorbance at 517 nm was measured, and the free radical scavenging activity was calculated according to Equation (2).

(2) $$\left(1-\frac{A_{sample}}{A_{control}}\right)\times 100\%=Radical\;scavenging\;activity(\%)$$

A_sample_ = absorbance of the sample

A_control_ = absorbance of the control

### 2.6. Statistical Analysis

OriginPro 2025b software (OriginLab, Northampton, MA, USA) was used to analyze the results statistically, and the data is expressed as the mean ± standard deviation (*n* = 3) for all tests.

## 3. Results and Discussion

### 3.1. Chemical Analysis

**Figure 1:**
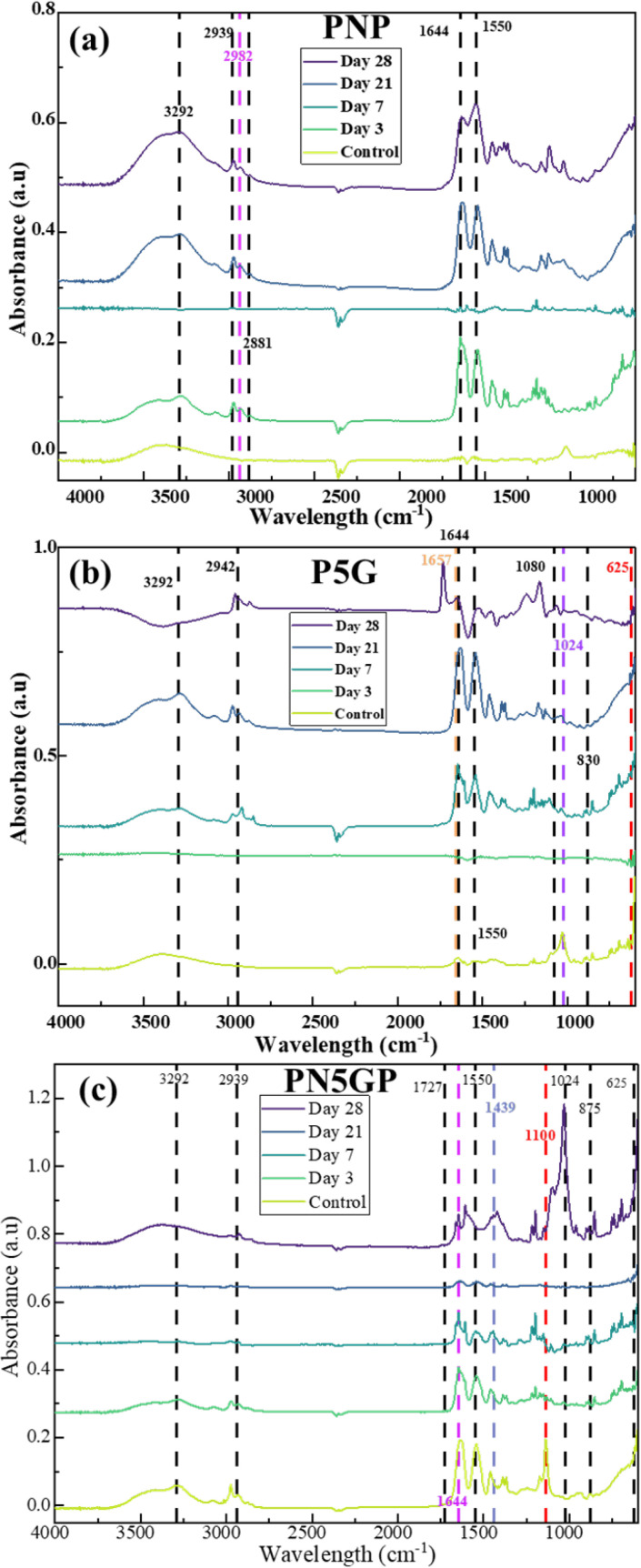
FTIR shows peak evolution over 28 days for (**a**) PNP, (**b**) P5G, and (**c**) PN5GP.

The FTIR spectra of the scaffolds at 0 (control), 7, 14, and 28 days reveal the chemical evolution of the matrix as degradation progresses (Figure 1).

Key peaks associated with PNIPAAm, PCL, and nHA were identified and monitored. The asymmetric CH_3_ stretching vibration, at approximately 2932 cm^−1^, from the isopropyl groups in PNIPAAm was present in all samples at day 0. It progressively diminished in intensity across all samples during the 28-day degradation period [25]. This decrease indicates cleavage of the polymer backbone and side chains, consistent with ester hydrolysis and chain scission [25,26]. The decline is most pronounced in PNP and PN5GP, suggesting higher degradation in these composites, while P5G retains the signal longer, reflecting improved matrix stability, likely due to GONS-induced reinforcement. The peaks around 3292 cm^−1^ correspond to the N-H stretching vibration of the amide group [25,26]. These peaks were present in all samples but were more pronounced in PN5GP at the start of degradation; however, through degradation, the peaks appeared less intense over time. The opposite trend was observed in PNP, where the peak was initially less noticeable but progressively increased in intensity through degradation. This peak’s intensity and position indicate changes in hydrogen bonding and hydration states [27]. These N–H moieties bond hydrogen with water molecules below the LCST, facilitating the reversible coil-to-globule transition that governs PNIPAAm’s volume phase change under physiological conditions [27]. The shifts show that PNP becomes more hydrophilic over time.

The carbonyl (C=O at 1725 cm^−1^) and C–O–C symmetric stretch (1160 cm^−1^) peaks, indicative of ester bonds, decreased sharply in PNP and PN5GP by day 28, confirming hydrolytic and oxidative degradation. P5G, however, showed a slower decline, attributed to GONS limiting water ingress and scavenging ROS.

Amide I (1650 cm^−1^) and II (1540 cm^−1^) peaks remained stable in P5G but diminished in PNP and PN5GP, highlighting the GONS’ protective role against oxidative cleavage. PN5GP’s rapid amide loss aligns with structural delamination and insufficient shielding by PCL. The Amide I band is indicative of the scaffold’s mechanical attributes and its capacity for metal coordination [28]. The carbonyl oxygen participates in hydrogen bonding, influencing scaffold elasticity, and is a ligand site for potential bidentate interactions with divalent metal ions, relevant in mineralization processes [29]. The increased intensity and shift of the Amide I peak are likely attributed to metal ion chelation with carbonyl groups and the initiation of mineralization processes, as Ca^2+^ coordinates with oxygen donors and facilitates calcium phosphate nucleation on the scaffold surface.

Phosphate peaks (1024 cm^−1^ and 625 cm^−1^) linked to nHA persisted or intensified in P5G and PN5GP, confirming mineral retention despite organic matrix degradation [30]. Progressive erosion of C–H, C=O, and C–O–C bands in PNP and PN5GP validated polymer fragmentation, while P5G’s attenuated degradation reinforced the GONS’ dual role as reinforcers and ROS scavengers.

### 3.2. Thermoresponsiveness and LCST

**Figure 2:**
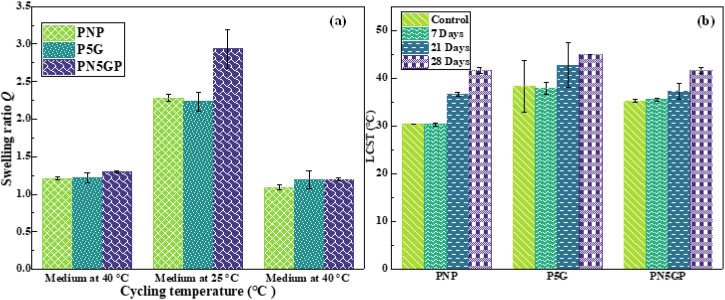
Graphs showing thermoresponsiveness of PNP, P5G, and PN5GP using (**a**) 12 h cyclical swelling (40 °C → 25 °C → 40 °C) and (**b**) LCST after fabrication and 7 and 21 days of oxidative degradation.

Swelling and deswelling responses to temperature changes reflect the scaffold’s ability to maintain structural functionality in fluctuating environments. Here, we assessed the thermoresponsive behavior of PNP, P5G, and PN5GP scaffolds through cyclic swelling experiments and LCST analysis before and after oxidative degradation (Figure 2a,b).

From Figure 2a,b, all the samples exhibit reversible swelling. At 25 °C, below the LCST for all the samples, swelling was observed, indicating that the networks were hydrated, whereas at 40 °C, they collapsed and expelled most of the water.

However, with repeated temperature cycling, thermoresponsive fatigue became evident, especially in PN5GP scaffolds. When first equilibrated at 40 °C, PN5GP showed a moderate swelling ratio (Q ≈ 1.4), which increased significantly to 2.9 upon cooling to 25 °C, due to enhanced water uptake facilitated by nanofiller-induced porosity. After reheating to 40 °C, however, Q dropped sharply to 1.1, indicating a loss in reversible swelling capacity. PNP also experienced the same phenomena. This irreversible collapse suggests structural rearrangement or pore closure within the PNIPAAm matrix. By contrast, P5G scaffolds maintained more consistent swelling behavior, showing minimal hysteresis and retaining higher Q values at 25 °C after cycling, likely due to more elastic and less constrained polymer networks.

Figure 2b also highlights the evolution of LCST in the PNIPAAm composites over the 28 days of degradation, taken at 7-day intervals. Initially, PNP had an LCST of 30 °C, slightly below the expected 32 °C, due to its specific network architecture or slight hydrophobic biases during polymerization [31]. P5G and PN5GP started with higher LCSTs, 38 °C and 35 °C, respectively, indicating a greater hydrophilic character out of the gate. The elevated LCST of P5G can be attributed to the highly hydrophilic nHA on the GONS and residual oxygenated groups on the GONS themselves, which increase the overall hydrophilicity of the scaffold, thereby requiring a higher temperature to trigger PNIPAAm collapse. PN5GP’s intermediate LCST suggests that the hydrophobic influence of PCL would lower the LCST, partially offsetting the hydrophilicity of the nHA-GONS, resulting in a net modest LCST increase compared to PNP [32,33]. Concurrently, P5G and PN5GP initially exhibit elevated LCSTs of 38 °C and 35 °C, respectively, which progressively increase to 42 °C and 37 °C throughout degradation. The increase in LCST signals an increase in the hydrophilic phase; this means that PNIPAAm’s degradation is dominated by oxidative chain scission [34].

**Scheme 2:**
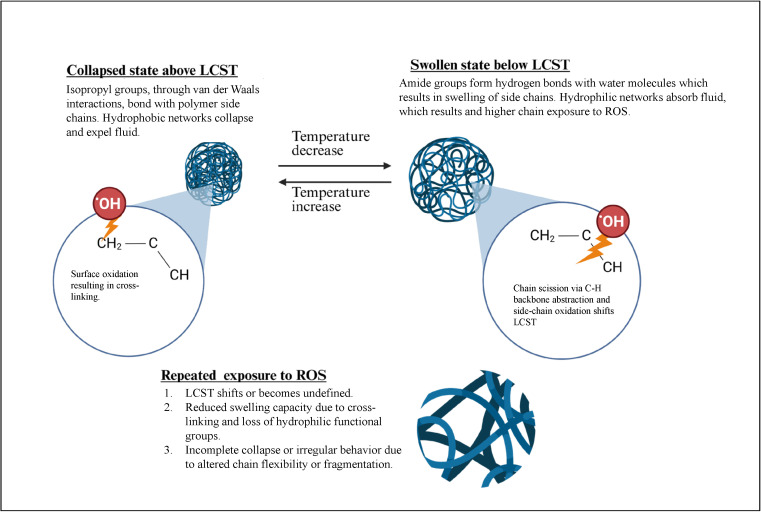
Schematic illustration of swelling and collapse of thermoresponsive scaffolds.

In PN5GP, introducing hydrophobic PCL domains created “locked” pockets of water; upon reheating to 40 °C, those regions prevented full de-swelling and thus reduced the subsequent re-swelling capacity. Incorporating rigid or hydrophobic phases can aggravate thermoresponsive fatigue by hindering complete water uptake [34]. Below the LCST, at 25 °C, the higher swelling of PN5GP (2.93) indicates that aggregation creates microscopic voids and cracks within the scaffold, which function as pathways for water to penetrate deeper into the material, increasing overall swelling capacity (Scheme 2).

The critical aspect of this study was determining whether compositing PNIPAAm with PCL microspheres would improve hydrolysis-driven chain relaxation degradation. The changes observed in PN5GP’s Q show that, with repeated fluctuations in temperature above and below the LCST, the composite absorbs less water, which could, in turn, mean reduced accessibility of ROS to polymer chains within the bulk of the scaffold. The addition of hydrophobic PCL disrupts this balance by forming rigid, water-repellent aggregates within the PNIPAAm matrix. PCL’s aggregation creates a paradox where high initial swelling accelerates hydrolysis, but structural collapse above the LCST, at 40 °C, isolates PCL regions, limiting sustained water access and masking long-term degradation effects. PNIPAAm reduces chain relaxation by retaining less water during repeated temperature cycling but sacrifices PN5GP’s swelling capacity. These swelling and deswelling trends are closely tied to each scaffold’s degradation mechanism and filler interactions, as evidenced by shifts in the effective LCST over time.

Chain scission drives the degradation process, and GONS mitigate oxidative degradation of PNIPAAm by neutralizing ROS, slowing the formation of hydrophilic groups [35]. The increases in LCST are slow but gradual, and each composite observes a different mechanism due to the role each nanofiller plays. The unprotected PNIPAAm undergoes rapid oxidative degradation in PNP, with LCST directly tracking chain scission.

In PN5GP, the LCST increases only modestly to 37 °C. This stability arises from PCL delamination, which exposes hydrophobic residues that counteract PNIPAAm’s oxidative hydrophilicity, delaying LCST rise. Over time, GONS may aggregate due to polymer erosion, reducing their hydrophobic sp^2^ surface area. This aggregation diminishes their LCST-lowering effect via π-π interactions with PNIPAAm, allowing the hydrophilicity-driven LCST rise to dominate oxidative degradation [35].

A study by Babu et al., aimed at thermally decomposing H_2_O_2_, showed that even at a pathology-relevant dose of 50 µL H_2_O_2_, the PNIPAAm-based hydrogels with manganese oxide nanoparticles still swelled over 24 h, demonstrating that PNIPAAm can operate as a real-time oxidative sensor, providing a warning that unprotected scaffolds will soften and fragment under chronic inflammation [36]. Mechanistically, changes in the LCST can be attributed to ROS abstracting backbone H-atoms, generating carbon-centered radicals that undergo β-scission. In parallel, side-chain oxidation converts the isopropyl groups on PNIPAAm to alcohols or ketones, resulting in increased hydrophilicity and a corresponding elevation in LCST [37].

Valencia et al. traced this LCST shift to the progressive oxidation of thioether functionalities into sulfoxides and sulfones, with the increase occurring almost linearly with the extent of oxidation [37].

Although LCST changes highlight the hydrophilic–hydrophobic balance governing thermoresponsiveness, mass loss profiles reveal telltale degradation kinetics driven by nanofiller. These kinetics differ from those reflected by the LCST, representing molecular-scale chain scission; mass loss quantifies bulk degradation and offers complementary data on how oxidative pathways proceed.

### 3.3. Mass Loss

**Figure 3:**
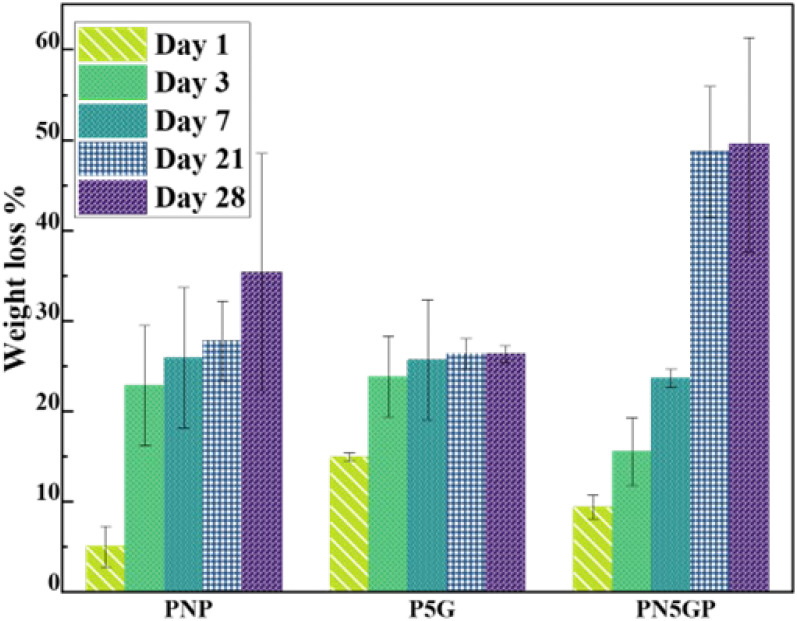
Percent mass loss of PNP, P5G, and PN5GP after 1, 3, 7, 21, and 28 days of oxidative degradation.

Scaffold degradation behavior under oxidative stress dictates long-term structural integrity and bioactivity. Mass loss profiles provide insight into the dominant degradation pathways, whether driven by hydrolytic chain scission, oxidative radical attack, or interfacial failure. We monitored mass loss in PNP, P5G, and PN5GP scaffolds over 28 days of oxidative immersion to correlate structural degradation with compositional design. Figure 3 highlights the effect of the oxidative degradation medium on the 3DP composite scaffolds.

PNP shows a steady increase in weight loss, with a big jump by day 3 and then a gradual increase up to day 28, while P5G starts with rapid degradation on day 1, then fluctuates slightly before stabilizing around day 21. On the other hand, PN5GP begins with moderate weight loss but shows a sharp increase after day 3, reaching 50% by day 21. The weight loss for P5G and PN5GP scaffolds stabilizes after day 21.

The weight loss patterns give a potential explanation for what happens to PNIPAAm as it degrades in the presence of nanofiller. At 30 °C, PNIPAAm transitions to a hydrophobic, collapsed state. At 37 °C, PNP remains collapsed, limiting bulk water penetration but allowing ROS to diffuse through the matrix. These ROS then dominate degradation, attacking PNIPAAm through radical-mediated pathways that slow initial weight loss [34].

P5G shows early degradation (15% by day 1) as ROS penetrate hydrophilic GONS interfaces, followed by fluctuations (~21% at day 7) due to the ROS-scavenging potential of GONS. By day 28, mass loss begins to plateau. In contrast, PN5GP displays delayed but exponential mass loss (~9% to 50% by day 28), dominated by delamination of PCL and PNIPAAm [38].

The sharp early degradation in PNP arises from ROS attacking PNIPAAm’s tertiary C–H bonds and amide groups, generating hydrophilic fragments (−COOH, −NH_2_) that enhance water uptake despite the scaffold’s collapsed hydrophobic state above 30 °C. Oxidative chain scission reduces molecular weight, accelerating mass loss. After 3 days, radical recombination, which is a termination reaction that occurs when two growing polymer radicals combine, results in cross-linking, forming a rigid network that impedes ROS diffusion and slows degradation [39].

The ANOVA results show that when comparing PNP, P5G, and PN5GP on each day, significant differences in mean weight loss were found on day 1 (*p* = 0.0010) and day 21 (*p* = 0.0025), indicating strong evidence of differing effects. In contrast, no significant differences were observed on day 3 (*p* = 0.1752), day 7 (*p* = 0.4955), or day 28 (*p* = 0.1350). When comparing days within each sample, PNP (*p* = 0.0085) and PN5GP (*p* = 0.0050) showed significant changes in weight loss over time, confirming a time-dependent effect, whereas P5G showed increased weight loss. There was no statistical difference across days (*p* = 0.3428).

The mass loss profiles underscore the distinct degradation pathways of the scaffolds, with P5G’s stabilized loss reflecting the GONS’ protective role, while PN5GP’s exponential loss signals structural failure from PCL-PNIPAAm’s phase separation. These degradation patterns likely influence the scaffolds’ surface morphology and capacity for biomineralization, which are essential for bone tissue integration.

### 3.4. Biomineralization and Surface Morphology

LAC-SBF is an effective medium to evaluate the physiological relevance of scaffolds, particularly their potential to bond with bone in vivo. In this study, SBF immersion tests revealed the mineralization potential across the three scaffold formulations, which correlated with their degradation kinetics and structural evolution.

**Figure 4:**
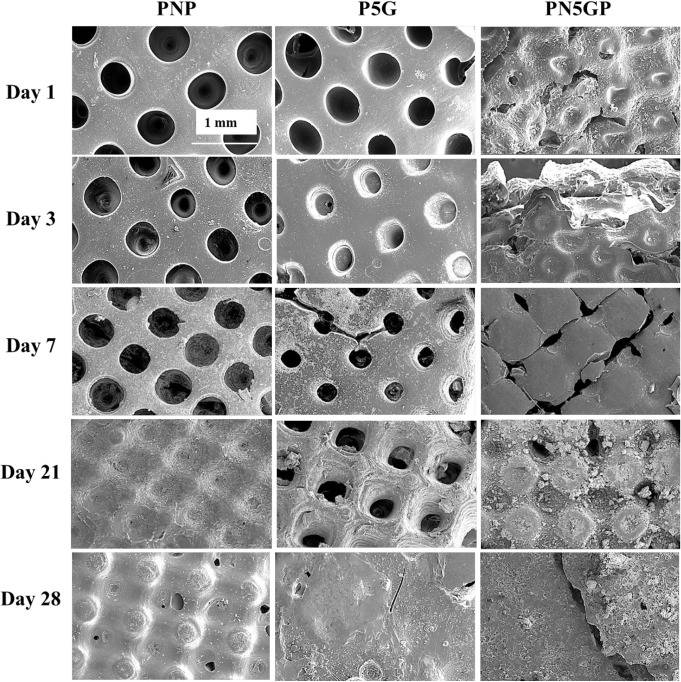
SEM images at 80× magnification of PNP, P5G, and PN5GP at 1, 3, 7, 21, and 28 days of oxidative degradation. Scale bar = 1 mm in all images.

Figure 4 shows that PNP starts with well-defined pores and some surface defects; however, by day 7, microspheres and cracks begin to form. These voids then lead to crack propagation and layer collapse over 28 days.

For PN5GP, no definitive pores are observed; these pores do not evolve, but eventually the layers collapse into each other. By day 7, severe layer separation and pore distortion are evident. This structural failure is primarily attributed to interfacial incompatibility and phase separation between PCL and PNIPAAm. The incompatibility arises because PNIPAAm undergoes significant volume phase transitions while PCL remains dimensionally stable, causing mechanical mismatch at the interface [27].

**Figure 5:**
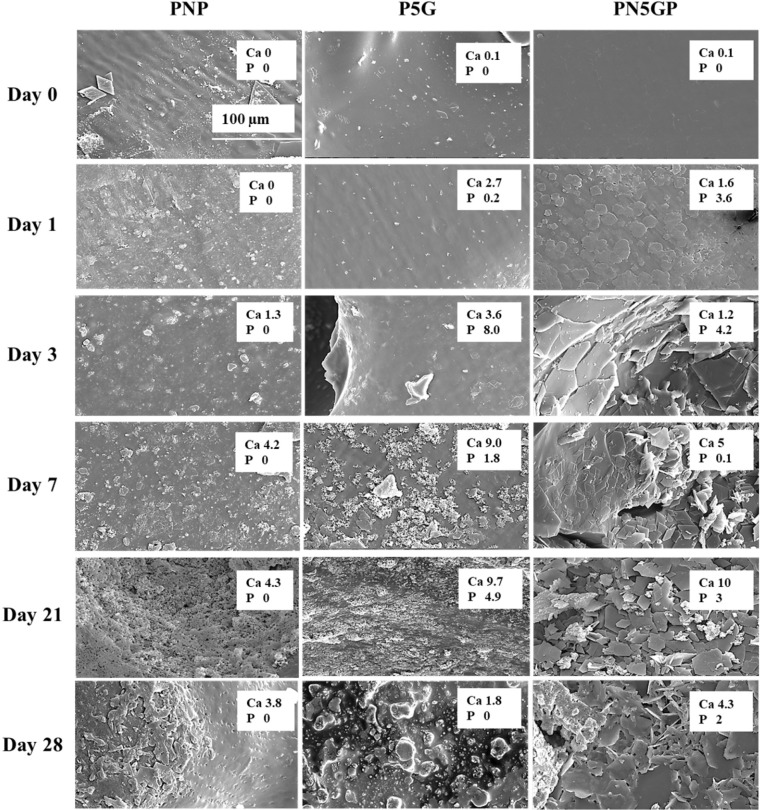
SEM images at 800× magnification showing Ca and P deposition of PNP, P5G, and PN5GP at 1, 3, 7, 21, and 28 days of oxidative degradation. Scale bar = 100 µm in all images.

Figure 5 shows mineralization of the scaffolds over the 28-day period. PNP’s surface shows some defects from fabrication.

However, a closer examination reveals microsphere formation from day 7 to day 28. On day 21, a mineral-like deposition is observed. On the other hand, P5G’s pore architecture remains relatively stable, with cracks and agglomerations observed from day 7 to 28. Finally, PN5GP starts with some roughness, but delamination occurs after 1 day of immersion.

Biomineralization trends across the scaffold formulations revealed mineral deposition patterns over the 28-day immersion period. Initially, PNP exhibited no detectable Ca or P; however, after day 3, sporadic increases in Ca only were observed. Ca/P deposition increased slightly across all groups up to day 28, reaching approximately 4.26% in the control. PN5GP scaffolds, however, exhibited reasonable mineralization, with competitive Ca/P increases by day 21, likely due to structural delamination that disrupted sustained surface deposition. Similarly, P5G scaffolds demonstrated more pronounced mineral accumulation, especially around the pore walls, reaching up to 10% Ca content by day 21 (Table 1).

Despite reasonable biomineralization, PN5GP showed delayed but exponential weight loss, from 22% to nearly 50% by day 21, consistent with the severe pore collapse and layer delamination evident in SEM micrographs (Figure 5). This delay suggests that structural degradation is not surface-limited but propagates throughout the bulk due to phase separation between PCL and PNIPAAm. FTIR further confirms this, with declines in C=O and C–O–C ester peaks (Figure 1c), indicating extensive hydrolytic and oxidative chain scission. The collapse of PN5GP pores is thus both a visual and molecular manifestation of scaffold degradation. In contrast, P5G showed early but stabilized mass loss, moderate structural changes, and slower chemical degradation, which is corroborated by retention of amide I/II peaks and a slower decline in ester peaks (Figure 1b).

**Figure 6:**
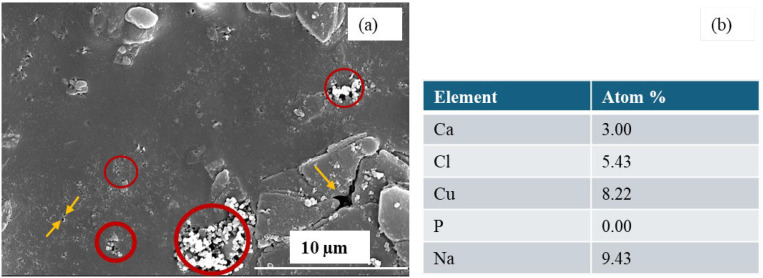
(**a**) SEM image at 8000× magnification showing microspheres. Microspheres are identified by the red circles and yellow arrows. (**b**) EDS ratios of Ca, Cu, and P deposition on PNP at day 21 of oxidative degradation.

In this study, Cu^2+^ was intentionally added to the SBF as CuSO_4_ to simulate oxidative stress conditions, and the detected copper on scaffold surfaces originates from this added source. However, a peculiar phenomenon observed in PNP is the deposition of Cu at days 21 and day 28 (Figure 6). Due to oxidative degradation at 37 °C, PNP exhibits microsphere formation because, above its LCST, it precipitates and gelates [27]. Additionally, the deposition of Cu is attributed to the introduction of carbonyl and carboxyl groups in PNIPAAm, which chelate Cu ions on the scaffold surface [40,41].

Furthermore, aggregated chains form dense microdomains or particles, which appear as microspheres shown in Figure 6a. Cu^2+^ chelates with PNIPAAm via coordination with amide groups along the polymer backbone, particularly under oxidative stress conditions that expose carbonyl and carboxyl functionalities. In this study, such chelation occurred in PNP scaffolds, where Cu^2+^ deposition was observed. While chelation is beneficial in applications such as metal ion sequestration, its occurrence here was unintentional. Chelated copper outcompeted Ca^2+^ deposition, limiting biomineralization. Moreover, excess copper accumulation poses cytotoxic risks, including inflammation and oxidative damage, further undermining scaffold biocompatibility [28,33,42]. Notably, Cu^2+^ deposition was not observed in PN5GP scaffolds, suggesting that GONS encapsulation and PCL phase separation limited copper ion access to chelating sites.

In PNP, each repeat unit offers a carbonyl oxygen and an amide nitrogen whose lone pairs readily coordinate Cu^2+^ in an O, N-bidentate fashion (Cu^2+^ simultaneously forming two bonds with the PNIPAAm) [43]. This type of bidentate coordination influences PNIPAAm’s chelation properties, so the ion can zip together adjacent chains and form stable chelate cross-links. In P5G, however, many of those same amide groups are already “busy”: they hydrogen-bond to epoxide and hydroxyl patches on the GONS’ surface, effectively masking their donor lone pairs. Because the PNIPAAm segments lie flattened against a curved, scroll wall, steric hindrance prevents the two donor atoms from wrapping simultaneously around the Cu center. The double penalty, isolation of donor electrons plus geometric crowding, which cuts accessible chelation sites and sharply lowers the overall Cu^2+^ uptake in the composite.

Copper uptake in the PN5GP microsphere scaffold is dictated by water accessibility and ligand strength in each microdomain. In the fully hydrated PNIPAAm matrix, Cu^2+^ diffuses freely and chelates through O, N-bidentate binding to amide carbonyls and nitrogen, so most of the copper that survives washing resides here, effectively cross-linking the hydrogel. At the PNIPAAm/PCL interface, the polymer chains flatten against the hydrophobic PCL surface, reducing chain mobility and partially hiding their amide sites; Cu^2+^ uptake is therefore very low.

The biomineralization and morphological analyses highlight P5G’s enhanced bioactivity and structural resilience, contrasted with PN5GP’s delamination and PNP’s unintended Cu deposition, driven by degradation and interfacial dynamics. A key factor in these outcomes is the scaffolds’ ability to mitigate oxidative stress, affecting structural integrity and bioactivity. To elucidate the role of GONS in combating ROS and sustaining scaffold performance, DPPH assays are paramount.

### 3.5. Antioxidant Potential

**Figure 7:**
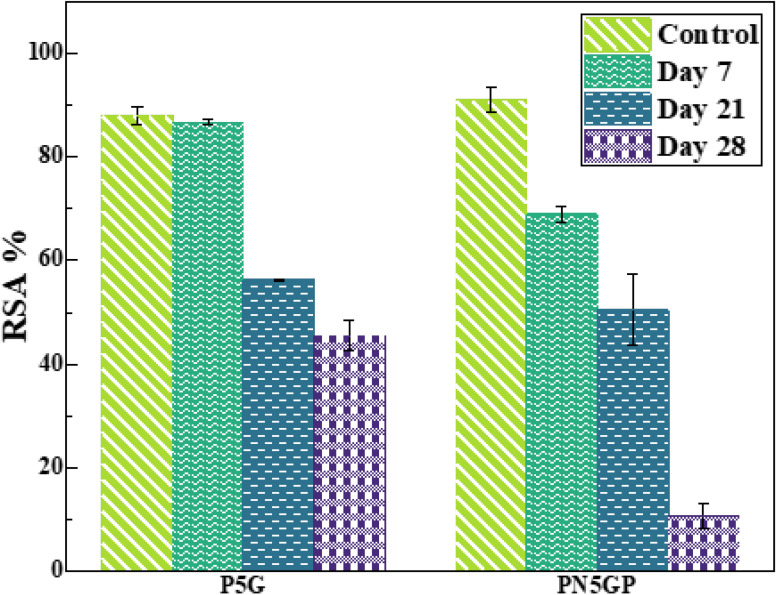
Graph tracking RSA % of P5G and PN5GP over 28 days.

The FTIR data and the DPPH-based radical scavenging results describe two sides of the same oxidative degradation coin: chemical changes recorded spectroscopically and the scaffold’s capacity to buffer those changes in real time. The radical scavenging activity (RSA) of P5G and PN5GP is shown in Figure 7 for day 0 through day 28.

Immediately after fabrication (day 0), the RSA of P5G and PN5GP was comparably high at 82 ± 2% and 85 ± 4%, respectively, confirming that the π-conjugated sp^2^ domains of GONS were fully accessible. By day 28, however, RSA in P5G had fallen to 45 ± 3%, whereas PN5GP retained only 16 ± 2%. However, RSA declined in both samples over 28 days, reflecting the progressive loss of active sites or shielding of the GONS’ surface during scaffold degradation.

PN5GP showed a significantly sharper decline, suggesting that its composite structure, incorporating both PCL and nHA-GONS, may limit the accessibility of GONS to ROS over time. The encapsulated design results in phase separation and interfacial disconnection, reducing water uptake and ROS contact at early stages, followed by delamination and degradation, disrupting the scavenging network’s continuity. In contrast, P5G retained a higher RSA after 28 days, indicating better GONS exposure and more stable antioxidant functionality. This suggests that although both scaffolds begin with comparable RSA, matrix design is critical in sustaining antioxidant activity during long-term oxidative degradation.

In PN5GP, the sharp fall of the C–H, C=O, and C–O–C ester bands at 1725 and 1160 cm^−1^ signals hydrolytic scission of the PCL backbone; once the π-conjugated surface of the embedded GONS is consumed, reflected by the rapid decline in RSA, those ester peaks collapse quickest. By contrast, P5G retains relatively steady ester and amide signals because the accessible GONS network quenches ROS, leaving 45% RSA even on day 28 and markedly slowing bond cleavage. The amide I/II envelopes at 1650/1540 cm^−1^ disappear fastest in PN5GP, implying that PNIPAAm domains delaminate from PCL, exposing fresh amide groups to unchecked oxidative attack while most GONS remain sequestered inside the PCL phase, unable to intercept radicals. Throughout the test, the phosphate vibrations of nHA (1024 and 625 cm^−1^) persist in both P5G and PN5GP, confirming the mineral’s chemical inertness; however, because nHA does not contribute to RSA, the antioxidant capacity plateaus once the GONS’ oxidant effect depletes.

Lu et al., 2018 reported that GO-reinforced ultra-high-molecular-weight polyethylene glycol (PEG) retained 68% of its initial carbonyl index after 12 months in SBF but suffered a hardness loss, attributing the accelerated decay to hindered GO accessibility within the hydrophobic polyethylene matrix [44]. PN5GP mirrors that behavior on a shorter, 28-day timeline, underscoring how matrix architecture, rather than GO content alone, governs oxidative resilience. Conversely, the 45% RSA retention in P5G edges out that of GO-PEG hydrogels, which held 40% RSA after 30 days, evidencing the benefit of keeping GONS thoroughly wetted inside a hydrophilic PNIPAAm network [45]. The higher residual RSA and slower bond-cleavage rate of P5G suggest that a simple PNIPAAm–nHA–GONS matrix can buffer inflammatory ROS across the critical four-to-six-week healing window, whereas the complex PCL/PNIPAAm hybrid of PN5GP risks early oxidative embrittlement. Improving PN5GP may require surface-grafting GONS onto the PCL phase in the PNIPAAm shell to restore continuous scavenger pathways. RSA depletes due to irreversible GONS oxidation compounded by matrix-dependent accessibility. PN5GP’s complex design accelerates depletion by isolating GONS, while P5G’s open network sustains scavenging by maintaining GONS exposure. These results confirm that RSA loss is not just chemical consumption, but a physical-access phenomenon governed by scaffold architecture. It should be noted that the results presented herein indicate the relative potential of the scaffolds to generate or interact with radical species under controlled in vitro conditions. While not directly predictive, these findings provide preliminary insight into how such antioxidant behavior may influence scaffold stability and performance in vivo, particularly under oxidative physiological conditions.

## 4. Conclusions

Adding PCL introduces a trade-off by enhancing residual hydration at 40 °C but sacrificing thermoresponsive reversibility. While P5G’s nHA-GONS optimize hydration without compromising reversibility, PN5GP’s dual-component system highlights the challenges of balancing hydrophilic/hydrophobic interactions. P5G exhibited the highest Ca and P deposition on day 28, indicating increased mineralization due to the templating effect of GONS. PNP exhibited low mineralization with Cu deposition after the stage of scaffold oxidation. PN5GP exhibited low ion retention, the cause of which was delamination and the absence of long-term surface exposure. The oxidative degradation of LAC-SBF/H_2_O_2_ led to composition-dependent weight loss and structural failure. For future development, interface optimization through GONS grafting onto PCL or nHA-GONS shell coatings could restore redox conduction pathways in PN5GP-like systems. These results position GONS as a multifunctional solution for SLA resins, with P5G excelling in oxidative environments and PN5GP offering a foundation for interface-optimized designs.

## Figures and Tables

**Table 1:** EDS elemental analysis showing calcium (Ca) and phosphorus (P) content, indicative of apatite formation on scaffold surfaces after immersion in SBF.

Sample	PNP		P5G		PN5GP	
	Ca (wt.%)	P (wt.%)	Ca (wt.%)	P (wt.%)	Ca (wt.%)	P (wt.%)
Day						
0	0	0	0.1	0	0.1	0
1	0	0	2.7	0.2	1.6	3.6
3	1.3	0	3.6	8	1.2	4.2
7	4.2	0	9	1.8	5	0.1
14	4.3	0	9.7	4.9	10	3
21	3.8	0	1.8	0	4.3	2
28	3.8	0	1.8	0	4.3	2

## Data Availability

The original contributions presented in this study are included in the article. Further inquiries can be directed to the corresponding author.

## Abbreviations

The following abbreviations are used in this manuscript:

ALP	Alkaline Phosphatase
ATR	Attenuated Total Reflectance
Ca	Calcium
Cu	Copper
CuSO_4_	Copper (II) Sulfate
DPPH	2,2-Diphenyl-1-picrylhydrazyl
DSC	Differential Scanning Calorimetry
EDS	Energy-Dispersive Spectrometry
FTIR	Fourier-Transform Infrared Spectroscopy
GO	Graphene Oxide
GONS	Graphene Oxide Nanoscrolls
H_2_O_2_	Hydrogen Peroxide
KCl	Potassium Chloride
LAC-SBF	Lactate-Modified Simulated Body Fluid
LCST	Lower Critical Solution Temperature
nHA	Nanohydroxyapatite
NIPAAm	N-isopropylacrylamide
P5G	PNIPAAm + 5 wt.% nHA-GONS
PCL	Polycaprolactone
PEG	Polyethylene Glycol
PN5GP	PNIPAAm + 5 wt.% nHA-GONS + PCL Microspheres
PNP	Pure PNIPAAm
PNIPAAm	Poly(N-isopropylacrylamide)
PBS	Phosphate-Buffered Saline
Q	Swelling Ratio
ROS	Reactive Oxygen Species
RSA	Radical Scavenging Activity
SBF	Simulated Body Fluid
SEM	Scanning Electron Microscopy
SLA	Stereolithography
sp^2^	sp^2^-Hybridized Carbon (conjugated carbon domain of graphene)
UV	Ultraviolet (used implicitly in photo-initiation context)
wt.%	Weight Percent
